# Is a good death possible in Australian critical and acute settings?: physician experiences with end-of-life care

**DOI:** 10.1186/1472-684X-13-41

**Published:** 2014-08-18

**Authors:** Steven A Trankle

**Affiliations:** 1 Centre for Health Research, School of Medicine, University of Western Sydney, Campbelltown, Australia

**Keywords:** End-of-life care, Intensive care, Palliative care, Good deaths, Qualitative research, Australia

## Abstract

**Background:**

In Australia approximately 70% of all deaths are institutionalised but over 15% of deaths occur in intensive care settings where the ability to provide a “good death” is particularly inhibited. Yet, there is a growing trend for death and dying to be managed in the ICU and physicians are increasingly challenged to meet the new expectations of their specialty. This study examined the unexplored interface between specialised Australian palliative and intensive care and the factors influencing a physician’s ability to manage deaths well.

**Method:**

A qualitative investigation was focused on palliative and critical/acute settings. A thematic analysis was conducted on semi-structured in-depth interviews with 13 specialist physicians. Attention was given to eliciting meanings and experiences in Australian end-of-life care.

**Results:**

Physicians negotiated multiple influences when managing dying patients and their families in the ICU. The way they understood and experienced end-of-life care practices was affected by cultural, institutional and professional considerations, and personal values and beliefs. Interpersonal and intrapsychic aspects highlighted the emotional and psychological relationship physicians have with patients and others. Many physicians were also unaware of what their cross-disciplinary colleagues could or could not do; poor professional recognition and collaboration, and ineffective care goal transition impaired their ability to assist good deaths. Experience was subject to the efficacy of physicians in negotiating complex bedside dynamics.

**Conclusions:**

Regardless of specialty, all physicians identified the problematic nature of providing expert palliation in critical and acute settings. Strategies for integrating specialised palliative and intensive care were offered with corresponding directions for future research and clinical development.

## Background

In Australia and the USA approximately 70% of all deaths are institutionalised but around 15% to 20% of deaths occur in intensive care settings where the ability to provide a “good death” is particularly inhibited [1-7]. Most Western countries, including Australia, struggle to provide palliative care in critical and acute hospitals [8]. Indeed, almost one quarter of those dying in ICU will spend their last days on a ventilator and almost 40% will die in pain [9,10]. Dying patients are often delivered to intensive care as a matter of procedure [11], but inappropriate settings, and efforts to meet unrealistic care goals, sometimes prescribe interventions that constrain the ability to manage deaths well [4,7,12]. Moreover, the emergency and acute nature of ICU can render planning difficult when patients sometimes deteriorate quite rapidly, and resourcing imperatives direct the focus of care to where life can be saved.

Technologically oriented, and staff intensive, the ICU is also an expensive area of health care [13,14]. For example, an Australian intensive care bed could cost as much as $3000-$4000 per day in comparison to a specialised palliative care bed at $600-$1600 per day depending on complexity of illness [15]. Importantly, palliative specialists in Australia argue that they can care for patients with wide ranging illnesses and multimorbidity not just the cancer patients that specialised palliative care has become synonymous with [16]. Many other cohorts of patients, like those with organ failure and neurodegenerative diseases, also require quality end-of-life care [4,16]. Additionally, a quality dying process must extend beyond the patient and include family and significant others and also consider those providing care. Many physicians find their skills and personal resources challenged when confronted by difficult situations that require a transition of care goals to palliation. For example, looking somebody in the eye and telling them they are dying is often quite disconcerting for physicians outside of dedicated end-of-life care settings [17-21]. Somewhat counterintuitively however, many palliative specialists are only consulted infrequently by their cross-disciplinary colleagues [4].

In order to provide appropriate end-of-life care that considers wide-ranging personal and professional interests, physicians need to negotiate many influencing factors [22-25]. For example, macro influences like politics, religion, and media; meso level influences such as medico-legal prescriptions, resourcing and profession-specific ideologies (e.g. comfort vs. cure) are brought to bear with the micro level influences of physicians such as their own personal beliefs, individual skills and moral/ethical positions. Physicians further engage with multicultural influences that include diversity in individual beliefs and attitudes of patients, loved ones and professional colleagues to make sense of practices.

Such complexity is important to consider when seeking to understand unique end-of-life care dynamics and the ability of physicians to assist good deaths. Furthermore, their efficacy in negotiating multiple influences at the bedside shapes their experiences [26]. Indeed, physicians experience burnout from personal [27-29] and also interrelated organisational influences [30,31], and research further identifies increasing numbers of physicians in Australia leaving their profession due to unsustainable demands being placed on them [32-35]. However, the complex interface of specialised intensive and palliative medicine, and connections between physician experiences and bedside outcomes, have not been explored in Australia.

Accordingly, the aim of this report is to document accounts of physicians in Australia who need to negotiate end-of-life care for patients and others in critical and acute settings. The broad-reaching interpersonal nature of managing death and dying is examined. Physicians’ experiences and their ability to assist “good deaths” are considered in relation to multiple contextualised influences.

## Method

This report draws from a larger body of qualitative research investigating end-of-life care, the purpose of which was to bolster limited knowledge, particularly in the Australian context. The problematic nature of palliation in critical and acute settings and the corresponding inconsistency in physicians’ practices and experiences, identified in an extensive literature review [36], was examined by addressing the research question: *How do physicians understand, negotiate and experience end-of-life care decision-making and practices in the context of Australian critical and acute settings?* Supportive research questions considered: *What is a good death? What influences a physician’s capacity to assist a good death?* Interview questions centred on personal experiences and individual beliefs and positions, good and bad deaths, and elicited information on context specific factors affecting how end-of-life care is provided (Appendix). However, a semi-structured approach allowed a great deal of physician direction and thus the potential for revealing other interesting paths of inquiry. In-depth interviews were one-on-one with the author and each between 60–90 minutes duration.

Prior to embarking on this research, approval was obtained from the University’s Human Research and Ethics Committee (# H7589). Letters of introduction, a research information sheet, and an informed consent document were generated for distribution to potential participants. A purposive recruitment strategy provided participants with relevant experience in end-of-life care, and across different clinical settings, with around half recruited through a snowball strategy after referral from their colleagues. Direct contact was made by the author to 16 physicians; 13 responded and agreed to participate. One was known personally to the author, some were selected through their professional register and others because of their published research. Sampling comprised seven palliative care specialists, three intensive care specialists, one respiratory/thoracic specialist with a major ICU role and two GPs. Participants were between 36 and 68 years of age, their experience in providing end-of-life care varied from six to over 40 years, and 8 physicians were male. Most practiced across the Sydney metropolitan area, two were from regional areas and two were from another capital city. The palliative specialists were engaged in both hospice and hospital settings. Due to the sensitive nature of the data physicians provided, and potential risks to their professional reputation, they were provided confidentiality and anonymity within legal boundaries. Each was given a pseudo-name, third party information was de-identified, and all interviews were transcribed exclusively by the author.

A thematic analysis, through an inductive/data driven approach, was conducted according to established guidelines [37]. Thematic analysis was compatible with the theoretical considerations of the research [24], and particularly useful for examining data that was previously unexplored. Correspondingly, the interpretive nature of analysis generated future research questions and directions.

As part of a larger body of research, trustworthiness was ensured by triangulation, which occurred through discussing the coding frame with my PhD advisory panel and arguing its rationale according to the data, and epistemological and theoretical approach grounding the study. I alone conducted coding, analysis and write-up; however, I sometimes drew guidance from my panel’s expertise in qualitative research and also for editorial comment. Further assisted through NVivo, an extensive series of notes and memos were generated to facilitate a process of checking and re-checking. These began by recording observational data and thoughts upon leaving interviews, and then throughout the analysis and written report. To ensure analytical rigor, deviant or discrepant cases were also evaluated. Data collection concluded when themes became saturated and nothing new emerged. It is also important to acknowledge that some researcher influence is unavoidable in any research design and particularly in qualitative research where interview dynamics have a large impact on the data obtained. Being aware of my reflexivity and documenting this, allowed me to attend systematically to every step of the research process. Finally, thematic and analytical integrity was assessed through successive chapter drafts leading to a completed thesis, which was accepted after examination. The following analysis presents and discusses selected key themes and their subthemes.

## Results and discussion

Three key themes were identified. The first two, *“Understanding a good death: Physicians’ perspectives”* and *“Whose job is it to Palliate: A changing system of care”*, concentrate on how physicians understand a good death and how they can practice end-of-life care from within critical and acute settings. Meaning is articulated. The remaining key theme, *“Negotiating appropriate care is a difficult experience”,* focuses tightly on the physicians’ corresponding experiences and recognises how the physician’s capacity to negotiate end-of-life situations, influences experiential aspects. Bolstered by a number of subthemes, the analysis illustrates the complexity in specialised end-of-life care.

### Understanding a good death: physicians’ perspectives

To assist a good death, it is important to understand how it is conceptualised by the physicians that provide end-of-life care. For example if physicians take a collaborative cross-disciplinary approach to care, then a common understanding in treatment goals and expected outcomes are essential. Although most aspects of a good death were identified with similarity among physicians, there were also specific aspects that reflected the individual nature of physician specialties and the settings they practiced from. A good death was described as multifaceted, but the idea of “peace and comfort” flowed through the physicians’ accounts.

#### Symptom control

All physicians, regardless of specialty, emphasised the importance of good symptom control. They often mentioned multimorbidity and symptomatic complexity that were crucial challenges in assisting a good death. Peace and comfort were frequently regarded as the desired goals and outcomes of symptom control. Physicians often described the importance of biopsychosocial considerations [26,38,39] where symptoms had multiple foundations and interactions. For example, Andrew (Intensive Care Specialist) said: *“wow that’s a broad question…if you ask me as a doctor, then I’d say, that’s a death that would involve…hopefully no pain at all, pain should always be managed…uhm, and wouldn’t involve too much anxiety”.* Similarly, Gina-Leanne (Intensive Care Specialist) considers a good death as a product of multiple factors that might interact symptomatically: *“a good death …would be one where they’re not in pain, they don’t have symptoms, they don’t feel distressed, they’ve had a discussion, they’ve had time to talk to their family and they feel…at peace with the decision”.* Reflecting holistic palliative ideals [40,41], she mentions a social aspect in conjunction with symptom control where time for family discussion may alleviate patient distress and provide peace for the dying patient and their family.

#### Planning for death (and care goal transition)

An ability to prepare for death and plan treatment options was important to most physicians. A good death was identified as one that was not unexpected, where appropriate care goal transition could take place and there was time to evaluate various treatment options. Sometimes this is problematic for physicians in critical/acute settings and in emergency situations where, for example, patients might be uncommunicative through intubation and sedation or by cognitive impairment [2,42,43]. Collaboration with patients and loved ones was emphasised by most physicians in deciding treatment goals. Time to prepare and plan allowed resources and strategies to be identified (and negotiated) that could assist a good death.

To illustrate, Andrew (Intensive Care Specialist) said: *“a good death…it shouldn’t come as a surprise to everyone, that there’s actually been some time…just to recognise that, the patient’s life is ending, and so there’s a plan in place; people know what they’re doing and what they’re treating”.* Andrew is also alluding to care goal transition here, where palliation may need to replace curatively focused interventions. But, he also considers planning with the family important: *“I feel that I’ve done a good job when I had the time to sit down with the family, there was a plan in place, everything was discussed and they recognised that this is the…proper way of doing things”.* Accomplishing a collaborative care plan minimises potential conflict by defining common goals, but it also brings Andrew some sense of achievement. He also mentions having the *time* to do this which reflects the busy nature of intensive care and its priority on saving life rather than managing death. Indeed, Aaron (Respiratory/Thoracic Specialist) also said: *“time is often against us and we struggle to get everyone on board with what we should be doing”.*

It is equally important to know when treatment withdrawal should be made, particularly when aggressive interventions are becoming futile, and when an appropriate and timely transition to a palliative model of care should occur. As Keith (Intensive Care Specialist) says: *“we need to know when to back off”.* At the same time, Gina-Leanne (Intensive Care Specialist) acknowledges the constraints in ICU that can inhibit a good death. She talks about care goal transition and patient transferal:

A good death is one in which we know the patient’s dying and we can get them to a stage where they can go somewhere else…to die, where there’s no restrictions on visiting hours, where they can have what they would like…do what they would like, which is just not possible in a busy ICU.

Gina-Leanne illustrates the pervasive ideology of curative goals in critical/acute settings that determine available resources (including time). She suggests that it is not always possible to know when a patient is dying when efforts are directed to saving life. She importantly identifies the ICU setting as an inappropriate place to die or receive end-of-life care. However, recognition of impending death is essential so that patients can be stabilised for transferral into appropriate care where they have more control. The timely transition of care goals cannot occur without preparation and Gina-Leanne considers discussion and planning as important aspects in a good death: *“A good death from an intensivist’s point of view is one that’s talked about, considered and planned”.* Death should not come as a shock.

#### Communication

Communication was regarded by most physicians as crucial in a good death. Gina-Leanne emphasised that a good death for an intensivist is “one that is talked about”. The ability to plan for death requires a capacity for communication. For dying patients and their family, communication and access to information are equally significant aspects in end-of-life care [44-46]. Communication between patients and loved ones provides a sense of closure, and peace and comfort. Indeed, Aaron (Respiratory/Thoracic Specialist) regards closure as important prior to death: *“being able to say goodbye is a very important part of the dying process”.* He believes communication between patients and their loved ones is essential at end-of-life, but that ability is not always possible: *“the family would not like their relatives to suffer, but they would also like some communication, some human contact rather than just sitting by the bedside with someone that’s comatose”.* Aaron emphasises meaningful human contact yet recognises communication may sometimes be problematic, where families occasionally wait for death beside their comatose loved one. There are suggested challenges to communication where great expertise is required to balance patient cognition with appropriate pharmacological symptom management strategies to avoided rendering patients comatose. Indeed, the issue of skilling up physicians across settings, or at least increasing access to specialist palliative expertise, is an important challenge and intensivists like Andrew acknowledge that: *“some of us are better at titrating than others”.*

With similar considerations, most physicians regarded information and the capacity for communication as principal goals in achieving a good death, particularly one of peace and comfort. Kerrie (Palliative Specialist) confirmed this: *“A good death is where the people around the deceased person…are comfortable. Families feel more comfortable when they have information, and know what’s going on, they have people to talk to, and see…the person looking calm and peaceful”.* Kerrie’s focus of care goes beyond the immediate patient and reflects the palliative ideology which considers and provides for broader care goals [40,41]. She emphasises a collaborative approach to end-of-life care that has the capacity to include family and others in discussions. Patients and their family, thus maintain some control over end-of-life outcomes.

#### Patient control (and input to end-of-life decisions)

Physicians stated that when patients have input into their treatment goals and decisions, a more comfortable and peaceful death might ensue. This was a major consideration by most (particularly palliative) physicians. For example, Jenny (Palliative Specialist) says: *“deaths are better if patients are involved in decisions”,* while Robert (Palliative Specialist) similarly suggests peace and comfort may be achieved if patients are able to retain some element of control:

I think a good death is…one in which the patient is reasonably at peace with the world, with their situation, and that their loved ones are reasonably comfortable with their journey as well…so with a patient at peace, and a part of that is people just feeling in some reasonable sort of control.

Robert regards end-of-life as a *journey* (both for patients and loved ones) illustrating palliative care ideals in which patients are not marginalised but rather encouraged to collaborate in decision-making and treatment goals [41]. Patient control enhances their sense of dignity, and mediates their suffering and fear of becoming dependent on others [47], but also requires having access to information on their illness. Physicians regard openness and transparency as important, but sometimes a good death means withholding information from dying patients, particularly if unable to convey it sensitively. Keith (Intensive Care Specialist) points this out:

A good death needs a system that’s open and transparent, while also being sensitive, and sometimes they [other physicians] don’t know what that means…like it’s easy to be perfectly blunt, but…sometimes, maybe you have to tell little white lies if you think they’re that sort of person.

Some physicians may not be skilled in sensitively communicating from within critical/acute settings, particularly when managing death is not the purpose of intensive care or the focus of training [7,14,48]. Unless this skill requirement is addressed, the level of control that patients have in directing decisions may remain at the discretion of individual physicians.

Yet many physicians also conceptualised a good death as to how it may apply to them and identified how important exercising personal control and input would be. For example, Peter (General Practitioner) said: *“I’d want control and options if it was me. That’s a good death, but I’ve seen many people trapped in a heartless system…suffering, with no way out…no capacity to direct treatment”.* Similarly, Aaron (Respiratory/Thoracic Specialist) said: *“a good death I think is someone who dies with dignity, who dies without suffering…some role in their own decision-making, so that’s…I think, what would be ideal for myself”.* Suffering and dignity are important humanistic considerations, but collaborative decision-making (that also includes families) may be difficult in Aaron’s specialty where patients are frequently intubated and ventilated. The technical nature of critical medicine more often places treatment decisions in the hands of physicians [49,50].

#### Existential considerations

Most physicians further considered addressing existential aspects important in a good death. Physicians understood a good death as one where a sense of meaning or purpose could be found, where life was fulfilling for the patient and things they considered important were taken care of. Indeed, Gary (Palliative Specialist) said: *“we help them look for what’s important to them now and after they’re gone”.* Existential considerations for physicians included looking beyond the disease or symptoms and regarding the person in the bed as valuable and important, including up to the moment of and beyond death. For example, Jeremy (Palliative Specialist) identifies his care focused on the *whole person: “I think part of helping death and dying is recognising the person in the bed, who they are, who they’ve been, and help them see the value they’ve been…that there’s a purpose, as much as that’s possible”.* Jeremy emphasises that end-of-life care is about helping patients find meaning in their life and maintaining their self-worth and value, and resisting discourses and practices that dehumanise them. Palliative models of care embody those ideals and goals, where empathy and dignity oriented care allow existential issues to be addressed [47,51,52].

Correspondingly, Andrew (Intensive Care Specialist) also acknowledges existential aspects: *“I mean a good death in a more general sense…is like; one where you would feel that person had fulfilled his or her life”.* However, in critical/acute settings such as Andrew’s, patients are often not aware they are dying [5,53,54]. They may often be sedated where cognitively they have little capacity to contemplate meaning of life, nor have time for this when intensive care, as curatively focused, often delays goal transition to palliative models of care [7]. An ability, therefore, to address existential needs, as important as they might be, may be determined by the setting and the patient’s condition.

### Whose job is it to palliate? A changing system of care

Patients often end up in ICU due to an acute episode of a chronic condition [4], or they are retained there, and specialist palliative expertise is difficult to access [54,55]. However, unlike palliative settings where death is actually expected and part of the management plan, palliation is not intended as part of the ICU brief. Yet, intensive care specialists need to manage death by default. Gina-Leanne illustrates the changing structure of intensive care, where a lot of people die and expectations are placed on specialist intensivists to palliate despite inadequate training and contrary motivations for entering their particular specialty: *“it’s not what people are trained in, not what people chose intensive care to do, we didn’t choose to be palliative care doctors, it’s just that…a lot of patients die when they’re with us and we have to do it”.* Andrew, another intensivist, believes death in end-of-life care should be appropriate to location, and planned: *“I think we really need to be doing a lot more education and forethought and thinking and planning and discussing, so that people don’t end up dying in ICU”.* ICU can be an inappropriate place to die and education and planning to allow death to occur elsewhere is crucial. Aaron (Respiratory/Thoracic Specialist) makes a similar point about working in the ICU: *“despite expectations that we can fix everything, people die here and many of us are unprepared for that…and it’s cruel inflicting invasive treatments on patients that shouldn’t be here when there are better places to die…we need broad-reaching education”.* Correspondingly, Keith (Intensive Care Specialist) described a “system” of care: 

Whenever we go around in intensive care, nurses and doctors, we often stop and say “don’t ever let that happen to me”… we generally feel as if the system’s taking this person too far - far, far too far; what the hell are they doing here, but it’s our job.

Given the choice, intensive care is not where physicians wish to die. Curative dynamics speak to a completely different focus of care, and these are pointed out by Andrew (Intensive Care Specialist): *“Well you’ve seen this place…it’s not a good place to…die, it’s noisy and busy, there’s…minimal privacy…there’s really no room for relatives to sit down and have some time by themselves”.* He further illustrates how he is forced in the ICU to prioritise care on those he can save, and those recognised as dying miss out: *“it’s so easy to have twenty other patients who are not meant for comfort measures only, who require your full attention and…I must, confess…that if it’s really busy we might neglect those patients for palliation…that’s obviously a very sad thing”.* Care goal transition to comfort models is problematic and often delayed. Yet care is reflective of a system that prescribes specific practices. Keith describes an end-of-life care process influenced at multiple levels. *“So patients, they’re just passed along, because of expectations of society, expectations of my colleagues, reluctance of my colleagues to discuss death and dying…it’s time consuming…they’re not comfortable with it, often…fear of litigation”.* A cross-disciplinary and collaborative approach to end of life care can be difficult to achieve and reflects how some deaths are subsequently managed. Indeed, Jenny (Palliative Specialist) spoke about poor symptom control but particularly how it is related to untimely goal transition and transfer to expert palliation:

A bad death to me is…the dying process hasn’t gone well, it’s somebody who’s, either referred…late, with uncontrolled symptoms, not aware of where they’re at… or they’ve been referred because their disease has been so rapidly progressing no one’s had a chance to get on top of things, no matter how much they’ve been trying, that’s the frustrating difficult and…very sad part.

As also identified by Robert (Palliative Specialist), poor care goal transition is a systemic issue where collaboration in common end of life care treatment goals can be elusive: *“…dealing with colleagues who…really, don’t value us as a specialty and…won’t refer people who could benefit…or… ignore our expert advice when we provide it, because of their own personal beliefs or their lack of knowledge”.* Similarly, Maggie (Palliative Specialist) said: *“we try so hard to raise our profile through advocacy, research and expert clinical practice, and still our specialty falls off the map”.* But as much as palliative recognition is problematic on the curative side of medicine, some who profess to advocate a comfort based approach to care do not further such goals effectively among other colleagues either. For example Kerrie (Palliative Specialist) said: *“it annoys me when some palliative people carry on as if they’re superior, they say things like ‘oh we wouldn’t do that-we’re the palliative care team’”.* Such attitudes may not be conducive to a collaborative relationship that optimises patient care and the efficacy of physicians across specialties to care for dying patients and their families.

The point of increased education and awareness is common in the excerpts physicians provide. As identified above, this certainly needs to take place at the cultural (macro) level to ensure that unrealistic expectations of cure are not perpetuated and that resources are applied appropriately. But clearly a similar emphasis needs to be made at the meso level where currently physicians across different specialties are unaware of what their cross-disciplinary colleagues can and cannot do. Efforts for enhancing an environment of greater collaboration are suggested, where the often unique needs of patients and their families can be effectively met more consistently and across settings. Physicians across specialties do recognise this where, for example, Kerrie (Palliative Specialist) said: *“we need to niche gentle subacute care across settings”* and, correspondingly, when canvassing the potential benefits of a palliative subspecialty within intensive care, Andrew (Intensive Care Specialist) said: *“yes that would be a great thing…a palliative-intensivist perhaps”.* In the USA, Hospice and Palliative Medicine has been approved as a subspecialty of 10 specialty medical boards but not for ICU and Critical Medicine [56]. This seems somewhat counterintuitive when, with similarity to the Australian situation, a significant number of deaths will occur in the ICU [2,4,5,44]. To 2011, the Centre to Advance Palliative Care (CAPC) reports almost 3000 US physicians across these specialty boards gaining palliative accreditation [56]. The experiences of physicians, patient families, and others involved in managing dying patients have benefitted with associated improvements in clinical practice. Skilling up physicians across settings in expert palliative techniques is also endorsed by Palliative Care Australia [57] yet, as evidenced by the present study, any such effort has not been successfully implemented.

Nonetheless, the evolution of intensive care in Australia need not necessarily threaten or pose insurmountable challenges for physicians who need to manage dying patients and their families. Indeed, accepting that death and dying will increasingly become the domain of ICU, Keith stands in contrast to many of his intensivist colleagues and draws satisfaction from the way his specialty is evolving:

“It’s a perverse direction we’ve taken in intensive care where people are increasingly brought to the ICU in the last few days of life…I mean I like saving lives, you know, getting people who are otherwise going to die, getting them alive again, that’s very satisfying still. But I must say that I get increasingly more satisfaction out of managing death…well”.

Certainly, Keith is subject to the constraints inherent in the ICU but he seems adaptive to the care requirements placed on him. Physicians like Keith who have great experience at the bedside, and who can attest to palliative efforts being professionally stimulating, can be great mentors and role models to others and help develop the changing face of intensive medicine. Some things are only learnt through experience. Indeed, when it comes to learning communication skills, Aaron (Respiratory/Thoracic Specialist) said: *“it’s probably a bit better these days but we were thrown in the deep end. You learnt to communicate on the job”,* and Keith said: *I’ve learnt tricks over the years, watching nurses and others. You know…like different ways of delivering the same message”.* As with programs in the USA seeking to integrate ICU and Palliative Care [58,59] and efforts to develop Leadership Centres for peer-to-peer mentoring utilising “local champions” [56], the bedside experience of some physicians is an extremely valuable asset that, if applied in the classroom, could benefit future (and current) generations of physicians who need to palliate in the ICU. Australian GPs have a mentoring program [60] but these programs are not widespread across specialties. Indeed, the lack of mentoring is emphasised by palliative specialists like Maggie who sometimes witness the needless suffering of patients:

I think it’s lack of knowledge and…attitudes…and just seeing what people without the clinical experience are doing…you know, hearing NFR conversations in the corridor…patients being dumped, but…half the time it’s through no fault of…the clinician…it’s just their lack of experience, or not having access to…experience and mentorship…but a lot of damage gets done and, we’re often in damage control…when we see someone in acute care.

### Negotiating appropriate care is a difficult experience

In a changing system of care, difficulties of palliating in the ICU are multitudinally influenced and strongly reflected in the experiences of physicians. This is an important recognition because a physician, who is restrained in providing care that best meets patient (and family) needs, is also at potential risk of burnout with all its adverse physical and psychological manifestations [27,28,31]. This is emphasised if a physician is also compelled to provide care inconsistent with their own personal and professional beliefs [61,62], or with inadequate training and resourcing. Such a physician, if unsupported, may be prone to making mistakes and/or disengaging from patients [31].

Although managing dying patients in the ICU is a positive experience for some intensivists like Keith, more often for most physicians it is a negative experience. Some physicians do not know what colleagues can and cannot do, and institutional constraints of resources and ideological prescriptions to care are additional factors physicians need to negotiate. Similarly, the diverse cultural beliefs and positions held by the wider community often play out inconsistently at the ICU bedside. Unrealistic expectations and an avoidance of death are engendered by a media that promotes the miracles of intensive care and medical technology [63], and a complicit system delivers patients to the ICU as a matter of process regardless how inappropriate [4]. Complex treatment decisions are often grounded in conflict at a time of great emotional difficulty.

#### Family conflict

The socially engendered discomfort of discussing death and dying, and accepting its inevitability [64-66], often became problematic for most physicians at the bedside. Expectations are placed on intensive medicine to cure and prevent death [5,13,67-69] and many physicians needed to negotiate a clinical or medical reality that conflicted with beliefs and attitudes held by families of dying patients.

Sometimes conflict with families and within families culminated in an aversive experience for these physicians and frequently translated into less than optimal care being provided for the patient (and the family). Andrew (Intensive Care Specialist) highlights this with some families applying pressure to inappropriately exhaust all options: *“some families push for everything to be done…and it’s, I mean it’s extremely distressing to see someone dying and knowing that there could be other ways of doing this”.* Andrew experiences such pressure as “extremely distressing”. He describes how his hands are tied but must witness a less than optimal dying process. Similarly, Aaron (Respiratory/Thoracic Specialist) also experiences conflict with the family over decisions aversively. He feels helpless when constrained from providing care in the patient’s best interests and describes such situations as “very, very difficult”, particularly when patients suffer needlessly or undergo futile treatment: *“I get a sense of helplessness…when families apply pressure…yeah, because you know what is the most appropriate way of managing the patient, uhm and to see someone suffering, or go through treatments, that’s not appropriate is very, very difficult”.* Conflicts over treatment decisions can, therefore, result in bad deaths, especially when opportunities for discussion or expressing final thoughts are lost. Jenny (Palliative Specialist) experiences these situations with distress: *“…until the day he died, there were barriers actually getting him comfort care…and that was distressing and inadequate, this person really didn’t have time to…for any issues to be discussed or…any last thoughts, I found that very distressing…a bad death”.*

The cultural diversity in Australian society fosters multiple opinions and attitudes that may conflict over clinical end-of-life decisions at the bedside. Gina-Leanne (Intensive Care Specialist) talks of some unhelpful cultural norms where sexism is considered acceptable. Her ability to provide appropriate care for a dying patient was impeded and consequently a negative experience for her, something she described as “horrible”:

Yeah I have experienced that (sighs) uhm…yeah, where you lose, well I didn’t have their trust ever from the word go because I was a woman and of course, in this particular culture “that’s a fat waste of time really”, uhm, you know “we want to speak to the Director” “well tough luck mate, you know I am the Director”… and uhm… yeah we lost their trust, and it was horrible, in the end we just… basically we had to sit there, and wait until the person’s, you know…brain, just about rotted.

In this relationship of conflict, Gina-Leanne was unable to assist the dying patient by withdrawing life support and letting them die. Families often wait for a miracle, sometimes unrealistically and will not accept medical evidence or the expertise of the physician [70] and, in some cultures, particularly if the physician is a woman [71]. Gina-Leanne found discriminatory dynamics in the relationship made developing trust difficult, something that is essential in end-of-life care [72,73]: *“I mean the worst thing you can do for a family is to lose their trust, once you’ve lost their trust…it’s just horrible…just horrible…”.* Multicultural requirements indicate that physicians need to be mindful of difference but also indicate a need for physician skills in difficult end-of-life negotiations, and support.

#### Collegial (Professional) conflict

Conflict is sometimes related to institutional and administrative structures that regulate where patients can be sent and how care may be provided. Care goal transition has been identified as problematic in the literature [2,7,44,54,74] and by most physicians in the present study. Patients need to be transferred into appropriate care as their condition develops, however this is also contingent upon others in the system accepting patients into their care. For example, Aaron (Respiratory/Thoracic Specialist) experiences professional conflict over patient transfer:

The major source of conflict that I have is basically intensive care. That’s the major problem I have. A patient in intensive care…someone decides look it’s useless, it’s hopeless, we need the bed for someone else, can we ship them up to your ward, and if you disagree with that, that’s where the conflict can occur…where most of my conflict, most of my difficulty is.

Aaron’s collegial difficulties are related to limited resources and receiving dying patients. He particularly identifies intensive care where there is little capacity for palliative efforts. Patients are subsequently forced elsewhere, to physicians like Aaron who might meet similar constraints for providing suitable care.

Yet many palliative specialists identified the lack of recognition their specialty receives from colleagues and how their expertise is frequently undervalued. Indeed, Maggie earlier described how her specialty “falls off the map”. Many palliative specialists identified how they often have capacity to take on critical patients transitioning to palliative goals despite the common perception among colleagues that palliative care is synonymous with cancer patients only [75]. Candice (Palliative Specialist) makes this point: *“we have many cancer patients but can handle most critical patients too, you know…hepatic, renal and cardiac patients…respiratory failures…end-of-life is all about multimorbidity…and it’s so frustrating and sad not being asked and seeing patients suffer who don’t have to”.* Professional etiquette also directs that physicians need to be asked. Candice is somewhat helpless and cannot just take over a patient who could benefit from her care. Similarly, Gary (Palliative Specialist) said: *“it’s so devastating watching people die bad deaths that could benefit from our help…we’re seldom given the chance…except by the oncologists”.*

Difficult clinical decisions with disagreement over treatment goals also frequently underpin professional conflict between physicians. Gina-Leanne (Intensive Care Specialist) talks of treatment withdrawal, describing how it is particularly problematic for her in the critical/acute setting:

Withdrawing is much more an issue with the cardiothoracic…patients; we would like to withdraw because we see it as futile, but the surgeons were very attached to their patients, the issue is that my colleagues will promise intensive care when it’s unwarranted…so they’ll have one of their patients they’ve looked after for a long time who’s slowly getting sicker and they’ll say “oh you know intensivists will do blah-blah” and, and invite us to go see them, the patient already has the expectation that we’re going to do blah and blah, and blah and blah is totally unwarranted. That really is a difficult problem for me!

Physicians in critical and acute care sometimes make unrealistic promises to patients and their families. Some surgeons have a considerable emotional investment in their patients and are reluctant to acknowledge that they are dying. She identifies how the societal expectation of intensive medicine to cure is further, but unhelpfully, promoted by colleagues. However, it places Gina-Leanne in a very difficult position between the reality of what her specialty can do and the unrealistic expectations of the patient and her colleagues. Andrew (Intensive Care Specialist) similarly points out how some physicians engender unrealistic expectations in their patients and patient families, and then hand them over to ICU. He experiences such instances with relatives of dying patients as “confrontational”: *“…mostly relatives, usually because they’ve been handled in the wrong way up until the point they have come in to us here, but expectations have been unreal, and, sometimes that can be very confrontational”.* Unrealistic expectations are promoted by an influential professional ideology that supports exhausting all resources in the hope of cure [5]. Understandably, relatives will cling to hope especially if it comes from the authority of a physician [76].

But some physicians have problems confronting a diagnosis of dying. Keith (Intensive Care Specialist) identifies medical peer pressure to save lives as a difficulty he experiences among colleagues: *“the constraints are medical peer pressure to cure…that’s the most difficult part”.* Again in curative settings, sometimes also through emotional investment where physicians might have developed a close relationship with patients, interventions are still aimed at patient survival and not comfort, as if death is a failure [77]. Indeed, Keith identifies with the uncertainty of some physicians when treating the dying and their motivation to incrementally increase treatment to cure and prevent death:

The other difficult part is ourselves in that your instinct tells you to stop…but we’re incrementally providing more and more treatment…they’re slipping away from us and we incrementally provide a bit more of this and a bit more of that and keep them…stable, so they, so they don’t slip away from us in the hope that they’ll turn around and get better. So it’s our uncertainty that’s another blocker.

Palliative specialist Jeremy acknowledges this also but, death is a given in his specialty: *“I teach students and try to help patients see that death is actually healthy and normal. Death is after all a given and not a medical failure, I believe that passionately…but my colleagues have struggled with this often”.* Jeremy challenges the idea of death as “medical failure” and illustrates how different ideological beliefs and prescriptions influence the physician’s experiences and capacity for managing dying patients.

#### Empathic connections and emotional pressure

End-of-life care can place significant emotional demands on the physician. As identified above, regardless of setting, physicians can be constrained by multiple factors in providing the best possible care. Interpersonal conflicts and difficulties, resourcing issues and ideological prescriptions directing care are examples. Correspondingly, the intrapsychic and uniquely personal of the physician is also a consideration at the bedside and interacts with these factors. For example, it can be assumed that most physicians have a capacity for empathy and compassion, but some physicians who are drawn to a specialty in end-of-life care are motivated to also develop strong communication skills and expertise with a comfort-based approach to care [14,48]. Although these physicians also experience adverse situations, and sometimes identify with particular patients, such negative experiences may be emphasised for physicians in the ICU who are not, as Gina-Leanne pointed out earlier, motivated or trained for this type of work. Death is not expected as it is in dedicated palliative or hospice units, and the skills and support a physician can draw on may be limited. Gina-Leanne particularly highlights the difficulty of communicating a diagnosis of dying: *“the hardest thing in ICU is to tell the patient that they’re dying, that there’s nothing else that you can do for them”.* Aaron (Respiratory/Thoracic Specialist) also said: *“it’s so hard when you’ve exhausted all options and you tell them that… they beg you ‘please…isn’t there anything you can do for me’”?*

A number of physicians also experienced similar emotional pressure, usually applied by family members of a dying loved one, to exhaust all avenues and save life. Not unexpectedly, this was something all ICU physicians experienced. For example, Keith said: *“families often expect miracles; they beg you to do something…that’s terrible sometimes…how do you tell them the brain-dead patient lying there will never come back to them”?* Similarly, Gina-Leanne talks of emotional pressure applied by loved ones who “bargain” with her to continue treating when withdrawing treatment is more appropriate:

One of the awful things that often comes up, is the issue of…say for example, bad head injury following trauma, we know there’s nothing more we can do for the patient, so ideally the best thing to do…would be to withdraw and let the patient die…and the families say “just one more day, just one more day, just one more day”…that makes it incredibly difficult when you’ve got a patient sitting there, you know it’s useless, futile, it’s a waste of time, but the family just want to hang on to them for another 24, 48 and they bargain with you, they’re not bargaining with you about, you know…a bag of groceries or something, their bargaining with you about somebody’s life!

Gina-Leanne is positioned as the one controlling life and death. It is “incredibly difficult” for her when loved ones “bargain” for the life of the dying patient. The constraints of culture and setting, which create expectations to save life, become a difficult obstacle that Gina-Leanne needs to negotiate. She acknowledges the dying person as not just a “bag of groceries” and highlights multileveled influences where socio-cultural engendered expectations of cure, and similar professional aspects, affect her experience of the physician/patient relationship.

### Limitations

Although this study provided unique insights into Australian end-of-life care practices and the experiences of physicians who manage dying patients and patient families in critical and acute settings, some research limitations merit consideration. Sampling was particularly appropriate for this qualitative investigation within the broader context of end-of-life settings, albeit with a potential self-selection bias. All palliative specialists had involvement with major hospitals and their critical/acute departments, however, the number of intensivists sampled is acknowledged as limited and, thus, further research is warranted. This has not negated the importance of documenting the findings. Of note, when physicians could manage deaths well they reported their experiences as positive but, more commonly, all of them regarded their experiences of negotiating the interface between palliative and critical/acute care as distinctly negative. This suggests that such experiences are more widespread among physicians elsewhere. Additionally, the present study accessed practicing physicians, yet many others with similar experiences who have ceased practicing for any multitude of reasons, some of which may include burnout, could also provide valuable data.

It is also important to acknowledge interview dynamics, with meaning and knowledge being “located” and a partly co-constructed effort, where no two interviews (even if repeated with the same interviewee) could ever be the same. Consistent with a complex and multidimensional world, there is more than one truth [22]. Accordingly, a more generalised research approach employing mixed methods and utilising characteristics of the present study could complement rather than diminish the present research.

## Conclusions

This study has identified how the current capacity for providing specialised end-of-life care in critical and acute settings in Australia is limited. Specialty specific approaches to care are mutually exclusive and influence the ability to provide “good deaths”. Curative goals are at odds with comfort-based approaches to care, yet physicians across specialties recognise the importance of integrating their efforts in common goals when managing dying patients and their families. A focus on the lived experiences of physicians has also demonstrated the importance of considering unique emotional and psychological factors, where the complexity of bedside interpersonal interactions influences outcomes. Patient and family care, and physician well-being and motivation, are all contingent on how specific and multitudinally influenced bedside dynamics can be negotiated. Physicians often experienced helplessness, sadness and distress, particularly when being compelled to practice against their better judgement and without adequate skills. They further identified poor collegial collaboration and support, where a lack of professional awareness and understanding of cross-disciplinary expertise often compounded institutional impediments to care.

This research was unique in the sense that comparable work has never been conducted in the Australian context, nor has any investigation considered the complexity within physicians’ end-of-life care practices and experiences. The research approach has allowed an in depth exploration of the multilevel influences involved in end-of-life care and has provided unique insights that may inform current and future efforts in Australia, and elsewhere, for integrating specialised palliative and intensive care. Indeed, capacities to qualitatively examine specificity and as many factors as possible can assist the clinical development of integrative care models especially where research findings are currently non-significant and/or conflicting. For example, after drawing on such models [58,59], some hospitals in the USA report increased palliative consults [78] and increased referral to specialised palliative care [79], while others report no reduction in length of ICU stay prior to death or in quality of dying [80]. However, the complex and contextually specific factors that uniquely influence the efficacy of integrative care models across settings have not been explored*.* A national approach to “skill up” physicians and enhance cross-disciplinary collaboration through similar integrative models can improve end-of-life care in Australian critical and acute settings, but their research and development must include macro, meso and micro considerations.

Integrating cross-disciplinary approaches to care may diffuse much of the conflict that physicians experience with their colleagues and also with patient families. Developing strong communication skills and enhancing access to mentoring and support, where physicians have a capacity to interact effectively with those from varied backgrounds, compliment other palliative skills they can bring to the bedside. The nature of critical care is changing but “good deaths” can become more common in these settings.

## Appendix

### Interview Schedule

Primary Research Question: *How do physicians understand, negotiate and experience end-of-life care decision-making and practices in the context of Australian critical/acute and palliative settings?*

Could you please tell me a little about your role in end-of-life (EOL) care?

– What is your background?

– What EOL settings do you have experience with (e.g. GP, palliative, critical, oncology, gerontology)?

– How long have you been providing EOL care in Australia?

Could I ask you, what do you see as a “good death”?

– Can you give me an example from your experience when a patient had a “good death”?

– What happened?

– What was that like for you? *(Explore deeper and seek a potential contrary)*

– What did you think about that?

– How did you feel about it?

– When are you not able to assist a good death? (*Explore setting/other constraints?)*

– Could you provide some examples?

– What suggestions could you make that would overcome setting barriers?

– Why?

– An example?

– How strongly does the institutional setting influence the care you provide?

– What do you see as a “bad death”?

– Could you describe an example from your experience in which a patient had a “bad death”?

– What happened?

– What was that like for you? *(Explore deeper)*

– What did you think about that?

– How did you feel about that?

Patient care for those with a terminal prognosis ultimately needs to progress from a critical or acute focus to one more palliative, for example, the cessation of more aggressive forms of treatment to a regime that emphasises comfort and palliation.

– Could you describe how you negotiate new care goals and treatment options with patients and their loved ones as they become necessary for patients with irreversible disease?

– What are some of the problems you encounter in such negotiations?

– Could you give me an example of a situation when you needed to do this?

– What happened?

– How did you feel?

Could I just ask you about your experiences with terminal or palliative sedation as an end of life practice?

– Could you give me an example where you used sedation with a particular patient?

– What happened in terms of a good or bad death?

– How did you feel on that occasion?

There may be times in end of life care when an intervention to alleviate suffering has the foreseeable but unintended consequence of hastening death. *(Increased analgesia-sometimes non-titrated due to restlessness; withholding antibiotics for pneumonia, withdrawing nutrition/hydration in PS etc.)*

– Could you give me an example from your experience when this occurred?

– What happened (in terms of a good or bad death)?

– What was that like for you?

– What were your thoughts about that at the time?

– How did you feel on that occasion?

– What other experiences have you had when using such end-of-life interventions?

– How have those experiences been different?

– OR

– When would you consider such interventions appropriate?

– What has been your experience with patients making a “specific” request for you to alleviate their *suffering* through death-hastening means?

– Could you describe a time when you received a patient request for assisted death?

– What happened?

– What did you do?

– What was that like for you?

– What were your thoughts about that?

– How did you feel on that occasion?

Research shows that suffering is often much more than physical, for example existential suffering, anxiety or fear over the progression of illness, loss of functional integrity and dignity, and loss of autonomy and independence, have been linked more strongly than physical pain to patient requests for hastened death.

– What has been your experience in receiving patient requests *(for death)* to end that kind of suffering?

– What happened?

– What did you do?

– What was that like for you?

– What were your thoughts about that?

– How did you feel on that occasion?

Have you had some experience with dying patients who, perhaps considering themselves a burden on others, felt it was their “duty to die”?

– Could you describe a time when you received a request to hasten death from a patient who considered it their “duty to die”?

– What happened?

– What was your response?

– How did you feel receiving such a request?

– Could you describe a time when you received a request from the patient’s family or loved ones to hasten a suffering patient’s death?

– What happened?

– What did you do?

– What was that like for you?

– What were your thoughts about that?

– How did you feel on that occasion?

Could you describe a situation where you considered making or would consider a decision to shorten the life of a dying patient who was suffering intractably?

– Could you provide an example?

– What was that like for you?

– What were your thoughts about that?

– How did you feel on that occasion?

When would you consider it appropriate (in terms of “good” or “bad” deaths) to “intentionally” hasten a patient’s death?

– What would it take?

– Are there any differences or exceptions?

Are there times when you experience a conflict between your ethical and professional duty to relieve suffering and your ethical and professional duty not to use means which deliberately hasten death?

– Could you describe a time when you had such a conflict?

– What happened? What did you do?

– How did you feel?

Are there times where you experience a conflict between your own personal standards and beliefs your ethical and professional duty to relieve suffering without hastening death?

– Could you describe a time when you had such a conflict?

– What happened? What did you do?

– How did you feel?

Could I just ask you a couple of final questions?

– Firstly, what’s the best part of your job?

– and not really wishing to end on a bad note, but what’s the worst part?

(End of interview)

Is there anything we might have missed or that came up in the interview that you would like to cover or talk further about?

## Competing interests

The author declares he has no competing interests.

## Authors’ information

Steven Trankle recently graduated from his PhD Candidature with the Centre for Health Research in the School of Medicine at the University of Western Sydney. His thesis was titled “End of Life Decisions and Practices: The Experiences of Doctors in Australia” [36].

## Pre-publication history

The pre-publication history for this paper can be accessed here:

http://www.biomedcentral.com/1472-684X/13/41/prepub

## References

[B1] Government of Victoria Australia Intensive care for adults in Victorian public hospitals 2003–04. Report to the public 2005 Australia: Victorian Government Department of Human Services 0 31

[B2] Beckstrand R Callister LC Kirchhoff K Providing a "good death": Critical care nurses' suggestions for improving end-of-life care Am J Crit Care 2006 15 1 38 46 16391313

[B3] Beckstrand R Kirchhoff K Providing end-of-life care to patients: critical care nurses’ perceived obstacles and supportive behaviours Am J Crit Care 2005 14 5 395 403 16120891

[B4] Hillman K End-of-life care in acute hospitals Aust Health Rev 2011 35 176 177 21612730 10.1071/AH10963

[B5] Hillman K Vital Signs: Stories From Intensive Care 2009 Sydney: University of NSW Press

[B6] Miles SH Randall-Curtis J, Rubenfeld GD The Role Of The Physician In Sacred End Of Life Rituals In The ICU Managing Death In The Intensive Care Unit: The Transition From Cure To Comfort 2001 Oxford: Oxford University Press 207 211

[B7] Seymour J O' Connor M, Aranda S Caring For Dying People In Critical Care Palliative Care Nursing: A Guide To Practice 2003 2 Melbourne: Ausmed Publications 329 339

[B8] Bloomer MJ Morphet J O'Connor M Lee S Griffiths D Nursing care of the family before and after a death in the ICU—An exploratory pilot study Australian Critical Care 2013 26 23 28 22309652 10.1016/j.aucc.2012.01.001

[B9] Meier DE Isaacs SL Hughes RG Palliative Care: Transforming The Care Of Serious Illness 2010 San Francisco: Jossey-Bass

[B10] Angus DC Barnato AE Linde-Zwirble WT Weissfeld LA Watson S Rickert T Rubenfeld GD Use of intensive care at the end of life in the United States: an epidemiologic study Crit Care Med 2004 32 3 638 643 15090940 10.1097/01.ccm.0000114816.62331.08

[B11] Hillman K Palliative Care Australia The Conveyor Belt Of Acute Care – What Should Happen? EoL Towards quality care at the end of life 2009 1 Australia: Spring 1 28

[B12] Afzal N Buhagiar K Flood J Cosgrave M Quality of end-of-life care for dementia patients during acute hospital admission: A retrospective study in Ireland Gen Hosp Psychiatry 2010 32 141 146 20302987 10.1016/j.genhosppsych.2009.10.003

[B13] Curtis JR Rubenfeld GD Managing Death In The Intensive Care Unit: The Transition From Cure To Comfort 2001 Oxford: Oxford University Press

[B14] Meier DE Beresford L Palliative care/intensive care unit interface: opportunities for mutual education J Palliat Med 2006 9 1 17 21 16430339 10.1089/jpm.2006.9.17

[B15] Education and Health Standing Committee Education and Health Standing Committee Destined To Fail: Western Australia’s Health System 2010 2 Perth: Legislative Assembly, Parliament of Western Australia 1 305

[B16] Palliative Care Australia Health System Reform And Care At The End Of Life: A Guidance Document 2010 1 90

[B17] Ahern SP Doyle TK Marquis F Lesk C Skrobik Y Critically ill patients and end-of-life decision-making: the senior medical resident experience Adv Health Sci Educ 2012 17 1 121 136 10.1007/s10459-011-9306-3 21626076

[B18] Moreno-Jiménez B Rodríguez-Carvajal R Hernández EG Benadero EM Terminal versus non-terminal care in physician burnout: the role of decision-making processes and attitudes to death Salud Ment 2008 31 2 93 101

[B19] Steinhauser KE Christakis NA Clipp EC McNeilly M Grambow S Parker J Tulsky JA Preparing for the end of life: Preferences of patients, families, physicians, and other care providers J Pain Symptom Manag 2001 22 3 727 737 10.1016/s0885-3924(01)00334-7 11532586

[B20] Back AL Arnold RM Baile WF Fryer-Edwards KA Alexander SC Barley GE Gooley TA Tulsky JA Efficacy of communication skills training for giving bad news and discussing transition to palliative care Arch Intern Med 2007 167 453 460 17353492 10.1001/archinte.167.5.453

[B21] Hancock KM Clayton JM Parker SM der Wal S Butow PN Carrick S Currow DC Ghersi D Glare P Hagerty R Tattersall M Truth-telling in discussing prognosis in advanced life-limiting illnesses: a systematic review Palliat Med 2007 21 507 517 17846091 10.1177/0269216307080823

[B22] Bhaskar R Reclaiming Reality: A Critical Introduction To Contemporary Philosophy 2011 London: Routledge

[B23] Potter G Lopez J After Postmodernism: An Introduction To Critical Realism 2001 London: Athlone Press

[B24] Williams SJ Beyond meaning, discourse and the empirical world: critical realist reflections on health Soc Theory Health 2003 1 42 71

[B25] Williams SJ Birke L Bendelow G Debating Biology: Sociological Reflections On Health, Medicine And Society 2003 London: Routledge

[B26] Adler HM Toward a biopsychosocial understanding of the patient-physician relationship: an emerging dialogue Soc Gen Intern Med 2007 22 280 285 10.1007/s11606-006-0037-8 PMC1824736 17357001

[B27] Keidel GC Burnout and compassion fatigue among hospice caregivers Am J Hosp Palliat Care 2002 19 3 200 205 12026044 10.1177/104990910201900312

[B28] Najjar N Davis LW Beck-Coon K Doebbeling CC Compassion fatigue: a review of the research to date and relevance to cancer-care providers J Health Psychol 2009 14 2 267 277 19237494 10.1177/1359105308100211

[B29] Sprang G Clark JJ Whitt-Woosley A Compassion fatigue, compassion satisfaction, and burnout: Factors impacting a professional's quality of life J Loss Trauma 2007 12 3 259 280

[B30] Girgis A Hansen V Goldstein D Are Australian oncology health professionals burning out? A view from the trenches Eur J Cancer 2009 45 393 399 19013790 10.1016/j.ejca.2008.09.029

[B31] Dunwoodie DA Auret K Psychological morbidity and burnout in palliative care doctors in Western Australia Intern Med J 2007 37 10 693 698 17517081 10.1111/j.1445-5994.2007.01384.x

[B32] Australian Medical Association ASMOFQ/AMA Queensland Safe Hours Report 2005 2005 Brisbane, Australia: AMA 1 40 Retrieved 15 May 2011 from http://www.amaq.com.au/docs/Safehoursreport.pdf

[B33] Australian Medical Association Joint statement - AMA, ADGP, RACGP, RDAA - Government's Medicare Package Courier Mail 2003

[B34] Coyne TJ Public or private: where would you choose to work? — Private Med J Aust 2011 194 9 949 10.5694/j.1326-5377.2011.tb03060.x 21534902

[B35] Australian Medical Association Safe Hours Audit 2006 2006 Canberra, Australia: AMA 1 11 Retrieved 31 May from http://ama.com.au/node/2492

[B36] Trankle SA End Of Life Decisions And Practices: The Experiences Of Doctors In Australia PhD Thesis 2013 Australia: University of Western Sydney http://trove.nla.gov.au/version/199976275

[B37] Braun V Clarke V Using thematic analysis in psychology Qual Res Psychol 2006 3 77 101

[B38] Engel GL The need for a new medical model: a challenge for biomedicine Science 1977 196 129 136 847460 10.1126/science.847460

[B39] Engel GL The clinical application of the biopsychosocial model Am J Psychiatr 1980 137 535 544 7369396 10.1176/ajp.137.5.535

[B40] Palliative Care Australia EoL – Towards quality care at the end of life Palliat Care Aust 2011 3 1 20

[B41] Saunders C Baines M Living with dying: the management of terminal disease 1983 Oxford: Oxford University Press

[B42] Barnes S Gott M Chady B Seamark D Halpin D Enhancing patient-professional communication about end-of-life issues in life-limiting conditions: A critical review of the literature J Pain Symptom Manag 2012 44 6 866 879 10.1016/j.jpainsymman.2011.11.009 22819438

[B43] DelVecchio GM Gadmer NM Ruopp P Lakoma MD Sullivan AM Redinbaugh E Arnold RM Block SD Narrative nuances on good and bad deaths: Internists’tales from high-technology work places Soc Sci Med 2004 58 939 953 14732607 10.1016/j.socscimed.2003.10.043

[B44] Curtis JR Rubenfeld GD Improving palliative care for patients in the intensive care unit J Palliat Med 2005 8 4 840 854 16128659 10.1089/jpm.2005.8.840

[B45] Clayton JM Butow PN Arnold RM Tattersall M Discussing end-of-life issues with terminally ill cancer patients and their carers: a qualitative study Support Care Cancer 2005 13 589 599 15645187 10.1007/s00520-004-0759-2

[B46] Clayton JM Hancock KM Butow PN Tattersall MHN Currow DC Clinical practice guidelines for communicating prognosis and end-of-life issues with adults in the advanced stages of a life-limiting illness, and their caregivers MJA 2007 186 12 S76 S108 10.5694/j.1326-5377.2007.tb01100.x 17727340

[B47] Chochinov H Dignity and the essence of medicine: the A, B, C, and D of dignity conserving care BMJ 2007 335 184 187 17656543 10.1136/bmj.39244.650926.47 PMC1934489

[B48] Billings ME Engelberg R Curtis JR Block SD Sullivan AM Determinants of medical students’ perceived preparation to perform end-of-life care, quality of end-of-life care education, and attitudes toward end-of-life care J Palliat Med 2010 13 3 319 327 20178433 10.1089/jpm.2009.0293 PMC2883506

[B49] Burgess DJ Dovidio J Phelan S Van Ryn M The effect of medical authoritarianism on physicians’ treatment decisions and attitudes regarding chronic pain J Appl Soc Psychol 2011 41 6 1399 1420

[B50] Pattison N A critical discourse analysis of provision of end-of-life care in key UK critical care documents Nurs Crit Care 2006 11 4 198 208 16869526 10.1111/j.1362-1017.2006.00172.x

[B51] Chochinov H Dignity-conserving care—A new model for palliative care: helping the patient feel valued J Am Med Assoc 2002 287 17 2253 2260 10.1001/jama.287.17.2253 11980525

[B52] Kissane DW Yates P O'Connor M, Aranda S Psychological And Existential Distress Palliative Care Nursing: A Guide To Practice 2003 2 Melbourne: Ausmed 229 243

[B53] Redpath R Parker J, Aranda S Negotiating New Goals And Care Options In The Presence Of Irreversible Disease Palliative Care: Explorations And Challenges 1998 Sydney: MacLennan & Petty 187 199

[B54] Le BHC Watt JN Care of the dying in Australia’s busiest hospital: Benefits of palliative care consultation and methods to enhance access J Palliat Med 2010 13 7 855 860 20636157 10.1089/jpm.2009.0339

[B55] Seymour J Negotiating natural death in intensive care Soc Sci Med 2000 51 8 1241 1252 11037214 10.1016/s0277-9536(00)00042-3

[B56] Robert Wood Johnson Foundation RWJF Program Results Report—Center to Advance Palliative Care 2012 1 37

[B57] Primary Health Care And End Of Life: Position Statement Position statement [http://www.palliativecare.org.au/Default.aspx?tabid=1942]

[B58] Nelson JE Bassett R Boss RD Brasel KJ Campbell ML Cortez TB Curtis JR Lustbader DR Mulkerin C Puntillo KA Ray DE Weissman DE Models for structuring a clinical initiative to enhance palliative care in the intensive care unit: A report from the IPAL-ICU Project (Improving Palliative Care in the ICU) Crit Care Med 2010 38 9 1765 1772 20562699 10.1097/CCM.0b013e3181e8ad23 PMC3267548

[B59] Nelson JE Azoulay E Curtis JR Mosenthal AC Mulkerin CM Puntillo KA Siegal MD Palliative care in the ICU J Palliat Med 2011 15 2 168 175 22280492 10.1089/jpm.2011.9599 PMC3388494

[B60] The RACGP Curriculum For Australian General Practice 2011 http://curriculum.racgp.org.au/statements/teaching,-mentoring-and-leadership-ingeneral-practice/

[B61] Festinger L A theory of cognitive dissonance 1957 Stanford, CA: Stanford University Press

[B62] Festinger L Conflict, decision and dissonance 1964 Stanford, CA: Stanford University Press

[B63] Drake M Cox P Ethics: End-of-life decision-making in a pediatric patient with SMA type 2: The influence of the media Neurology 2012 78 23 e143 e145 22665147 10.1212/WNL.0b013e318258f835

[B64] Kellehear A Morgan JD, Laungani P The Australian Way Of Death: Formative Historical And Social Influences Death and bereavement around the world Volume 4: Death and bereavement in Asia, Australia and New Zealand 2005 New York: Baywood Publishing Company 11 22

[B65] Kellehear A The Study Of Dying. From Autonomy To Transformation 2009 Cambridge, UK: Cambridge University Press

[B66] Mills S Discourse 2004 2 New York: Routledge

[B67] Curtis JR Vincent JL Ethics and end-of-life care for adults in the intensive care unit Lancet 2010 375 1347 1353 20934213 10.1016/S0140-6736(10)60143-2

[B68] Robichaux CM Clark AP Practices of expert critical nurses in situations of prognostic conflict at end-of-life Am J Crit Care 2006 15 5 480 489 16926369

[B69] Stolick M Dying to meet you: Facing mortality and enabling patient styles Am J Hosp Palliat Care 2003 20 4 269 273 12911071 10.1177/104990910302000408

[B70] Widera EW Rosenfeld KE Fromme EK Sulmasy DP Arnold RM Approaching patients and family members who hope for a miracle J Pain Symptom Manag 2011 42 1 119 125 10.1016/j.jpainsymman.2011.03.008 21641763

[B71] Glick P Fiske ST Mladinic A Saiz JL Abrams D Masser B Adetoun B Osagie JE Akande A Alao A Brunner A Willemsen TK Chipeta K Dardenne B Dijksterhuis A Wigboldus D Eckes T Six-Materna I Exposito F Moya M Foddy M Kim H Lameiras M Sotelo MJ Mucchi-Faina A Romani M Sakalh N Udegbe B Yamamoto M Ui M Beyond prejudice as simple antipathy: Hostile and benevolent sexism across cultures J Pers Soc Psychol 2000 79 5 763 775 11079240 10.1037//0022-3514.79.5.763

[B72] Mack JW Block SD Nilsson M Wright A Trice E Friedlander R Paulk E Prigerson HG Measuring therapeutic alliance between oncologists and patients with advanced cancer: The Human Connection Scale Cancer 2009 115 14 3302 3311 19484795 10.1002/cncr.24360 PMC2771331

[B73] Heyland DK Dodek P Rocker G Groll D Gafni A Pichora D Shortt S Tranmer J Lazar N Kutsogiannis J Lam M What matters most in end-of-life care: Perceptions of seriously ill patients and their family members Can Med Assoc J 2006 174 5 627 633 16505458 10.1503/cmaj.050626 PMC1389825

[B74] St-Laurent-Gagnon T Carnevale FA Duval M Pediatric palliative care: a qualitative study of physicians' perspectives in a tertiary care university hospital J Palliat Care 2008 24 1 26 30 18459594

[B75] O'Leary N Tiernan E Survey of specialist palliative care services for noncancer patients in Ireland and perceived barriers Palliat Med 2008 22 1 77 83 18216080 10.1177/0269216307084609

[B76] Curtis JR Engelberg R Young JP Vig LK Reinke LF Wenrich MJ McGrath B McCown E Back AL An approach to understanding the interaction of hope and desire for explicit prognostic information among individuals with severe chronic obstructive pulmonary disease or advanced cancer J Palliat Med 2008 11 4 610 620 18454614 10.1089/jpm.2007.0209

[B77] Buxton D The intern experience: facing death J Palliat Med 2011 14 6 784 786 21651369 10.1089/jpm.2010.0512

[B78] Sihra L Harris M O'Reardon C Using the improving palliative care in the intensive care unit (IPAL-ICU) project to promote palliative care consultation J Pain Symptom Manag 2011 42 5 672 675 10.1016/j.jpainsymman.2011.08.002 22045371

[B79] O'Mahoney S McHenry J Blank AE Snow D Karakas SE Santoro G Selwyn P Kvetan V Preliminary report of the integration of a palliative care team into an intensive care unit Palliat Med 2010 24 2 154 165 19825893 10.1177/0269216309346540

[B80] Curtis JR Nielsen EL Treece PD Downey L Dotolo D Shannon SE Back AL Rubenfeld GD Engelberg RA Effect of a quality-improvement intervention on end-of-life care in the intensive care unit: a randomized trial Am J Respir Crit Care Med 2010 183 348 355 20833820 10.1164/rccm.201006-1004OC PMC3056230

